# Virus-Induced Gene Silencing (VIGS) in *Hydrangea macrophylla* and Functional Analysis of *HmF3′5′H*

**DOI:** 10.3390/plants13233396

**Published:** 2024-12-03

**Authors:** Qiyu Yang, Youwei Fan, Shuwen Luo, Chun Liu, Suxia Yuan

**Affiliations:** State Key Laboratory of Vegetable Biobreeding, Institute of Vegetables and Flowers, Chinese Academy of Agricultural Sciences, Beijing 100081, China

**Keywords:** *Hydrangea macrophylla*, VIGS, *HmPDS*, *HmCHS1*, *HmF3′5′H*

## Abstract

*Hydrangea macrophylla*, renowned for its large inflorescences and a diverse range of colors, highlights a significant limitation in current gene function research, which is the lack of effective molecular genetic tools. This study utilized a *tobacco rattle virus* (TRV)-based virus-induced gene silencing (VIGS) system to investigate gene function through posttranscriptional gene silencing in *H. macrophylla* for the first time. The ortholog of *phytoene desaturase* (*PDS*) in *H. macrophylla*, termed *HmPDS*, was identified. Infection of tissue-cultured seedlings with TRV-*HmPDS* led to photobleaching of the leaves. Additionally, infection with TRV containing the *HmCHS1* fragment in the flowers resulted in decreased anthocyanin production in sepals and a lightening of sepal coloration in the infected flowers. The phenomena and RT-qPCR results proved that the *PDS* and *CHS* genes of hydrangea were successfully silenced via the vacuum infiltration method. Furthermore, the introduction of TRV-*HmF3′5′H* revealed a decrease in delphinidin-3-glucoside content in sepals and caused a color change in the sepals from blue to pink. This study demonstrated that the TRV-VIGS system was successfully established in *H. macrophylla* and effectively applied to the function analysis of *HmF3′5′H*.

## 1. Introduction

*Hydrangea macrophylla* is an important ornamental plant with significant commercial value as potted, cut, and dried flowers. Additionally, it is highly favored by consumers for its huge flower clusters, vibrant flower colors, diverse varieties, and extended blooming duration. Furthermore, *H. macrophylla* is commonly cultivated in gardens due to its strong adaptability, resistance to diseases and pests, and tolerance to cold and shade. Additionally, research has indicated that *H. macrophylla* possesses various medicinal properties [1,2,3]. Nevertheless, functional genomic research remains constrained due to the absence of efficient molecular genetic tools. Therefore, it is imperative to develop tools for elucidating gene functions within *H. macrophylla*.

Virus-induced gene silencing (VIGS) technology harnesses a recombinant viral vector that includes a fragment of a target plant gene to activate RNA-mediated post-transcriptional gene silencing, resulting in the suppression of transcripts of homologous genes [4,5]. Comparison to gene silencing techniques that utilize inverted repeat sequences, VIGS presents several benefits, such as simpler plasmid construction, a brief operational period, and the ability to identify embryo-lethal genes [6]. Recently, a *Tobacco rattle virus* (TRV)-based VIGS technology has been applied to various ornamental plants to verify gene function, such as *Paeonia ostia* [7], *Rosa* sp. [8], and Lily [9]. VIGS is increasingly acknowledged as a highly valuable tool for exploring gene function. A key advantage of VIGS is its ability to produce rapid phenotypes without requiring stable plant transformation [10]. In comparison to other methods, including T-DNA, transposon insertion techniques, chemical and physical mutagenesis, and functional genome editing approaches like CRISPR-Cas, VIGS is a cost-effective option [11]. However, no reports have yet documented the successful use of TRV in *H. macrophylla* for gene silencing.

In this study, we aim to establish a VIGS system in *H. macrophylla* and use it for the functional validation of the *HmF3′5′H* gene. This VIGS system will be employed for the functional characterization of hydrangea genes in future studies.

## 2. Results

### 2.1. Identification of HmPDS

**Figure 1:**
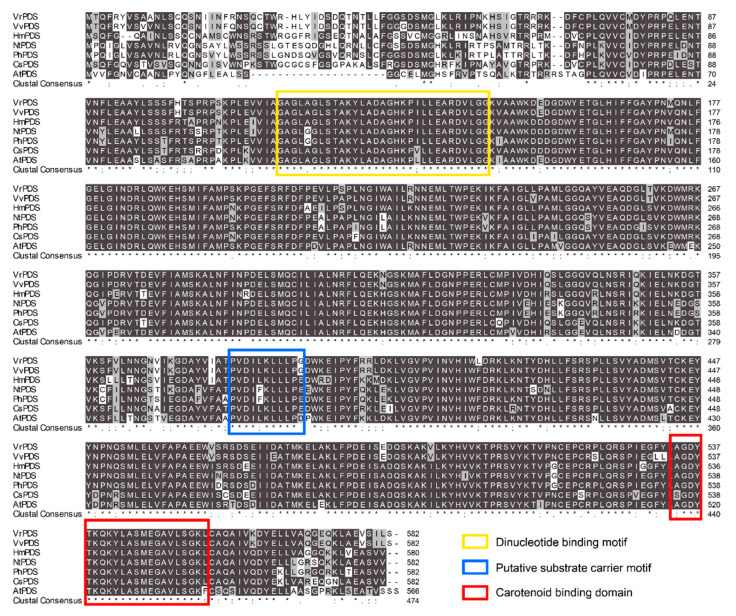
Multiple sequence alignment of deduced amino acids of HmPDS with other homologies. Colored boxes on amino acids indicate the position of motifs and domains. The asterisk means that the residue is the same, and the dot means that the residue is conserved. Homologous sequences from other plant species included VrPDS (KAJ9691772.1) in *Vitis riparis*, VvPDS (AFP28796.1) in *Vitis vinifera*, NtPDS (BCG50296.1) in *Nicotiana tabacum*, PhPDS (AOG29531.1) in *Petunia hybrida*, CsPDS (KAF5947778.1) in *Camellia sinensis*, and AtPDS (AAL15300.1) in *Arabidopsis thaliana*.

The ORF nucleotide sequence of *phytoene desaturase* (PDS) from *H. macrophylla*, referred to as *HmPDS*, was amplified by PCR and annotated based on transcriptome data. *HmPDS* was predicted to encode a protein comprising 581 amino acids with three motifs, including a dinucleotide binding motif, a putative substrate carrier motif, and a carotenoid binding domain. The sequence of *HmPDS* was submitted to the NCBI database under accession number GenBank: PQ285401. Multiple sequence alignments revealed the amino acid sequence of HmPDS exhibited identities that varied from 82.4% to 85.9% with homologous sequences identified from six other plant species (Figure 1).

### 2.2. Silencing of the PDS Gene in Plantlets of H. macrophylla

**Figure 2:**
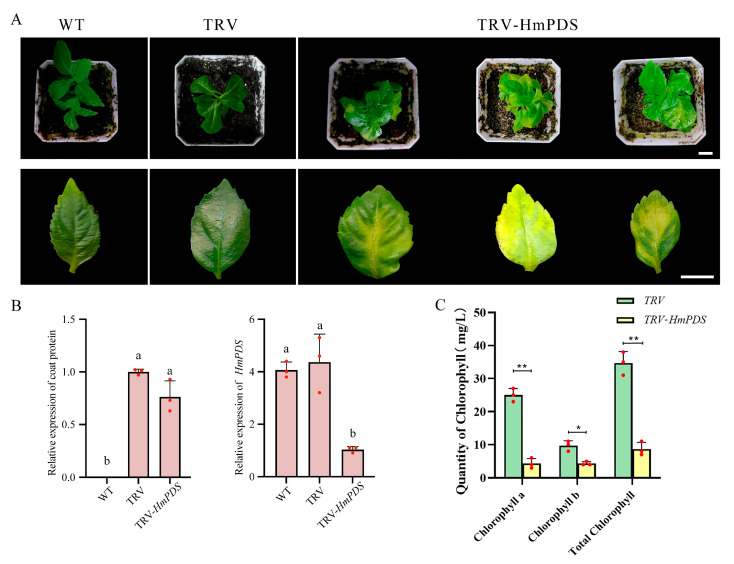
TRV-induced *PDS* silencing in *H. macrophylla*. (**A**) Morphological comparison of wild-type plantlet (left), plantlets infected with TRV (middle), and TRV-*HmPDS* (right). The photos were taken on day 30 after infiltration. White scale bar equals 1 cm. (**B**) RT-qPCR analysis to detect transcripts of TRV and *HmPDS* in leaves under various treatments. Different lowercase letters indicate significant differences at *p* < 0.05 as determined by Duncan’s test. (**C**) Comparison of the content of chlorophyll a, chlorophyll b, and total chlorophyll between *PDS*-silenced and TRV-infected plantlets. Samples were harvested on day 30 after the infiltration. Asterisks indicate statistically significant differences between TRV-*HmPDS* and TRV control (Student’s *t*-test, * *p* < 0.05, ** *p* < 0.01). Values were the means ± standard deviation (SD) of three independent biological replicates (*n* = 3).

The TRV-*HmPDS* recombinant was designed to target the endogenous *PDS* gene in leaves of *H. macrophylla* via vacuum infiltration. Thirty days post-infection, 60% of the TRV-*HmPDS*-infected seedlings displayed a photo-bleached phenotype (Figure 2A), whereas control plantlets infected with TRV retained green coloration. The presence of TRV in hydrangea was confirmed by RT-qPCR with primers specific to the TRV2 coat protein. The expression of *PDS* was significantly reduced in TRV-*HmPDS*-infected plantlets compared to the TRV-infected controls (Figure 2B). Furthermore, the levels of chlorophyll a, chlorophyll b, and total chlorophyll were all decreased in plantlets with *HmPDS* silencing (Figure 2C). These results demonstrate that *HmPDS* in plantlets of *H. macrophylla* can be effectively silenced using the VIGS system.

### 2.3. Evaluation of a VIGS System in H. macrophylla Flowes

**Figure 3:**
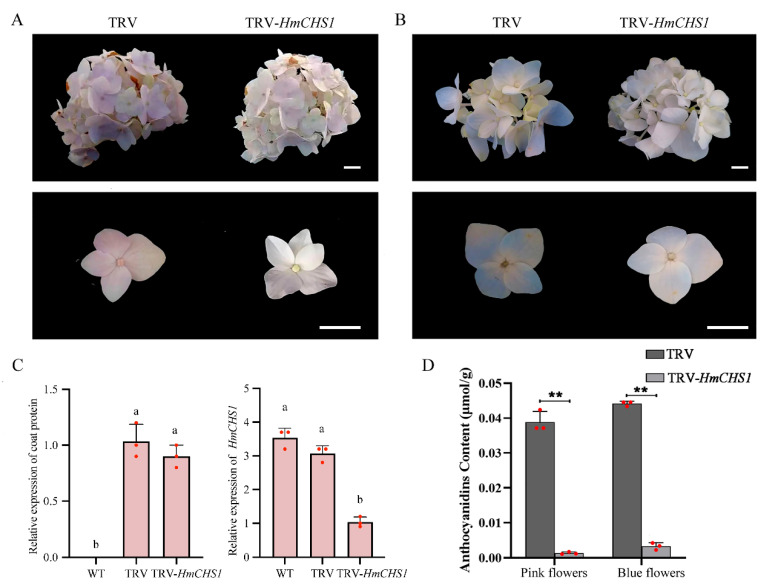
TRV-induced *CHS* silencing in *H. macrophylla*. Morphological comparison of (**A**) pink flowers and (**B**) blue flowers infected with TRV and TRV-*HmCHS1*. Photos were taken on day 15 after infiltration. White scale bar equals 1 cm. (**C**) RT-qPCR analysis to detect transcripts of TRV and *HmCHS1* in flowers under various treatments. Different lowercase letters indicate significant differences at *p* < 0.05 as determined by Duncan’s test. (**D**) Comparison of the content of total anthocyanidin between *HmCHS1*-silenced flowers and TRV control. Asterisks indicate statistically significant differences between TRV-*HmCHS1* and TRV control (Student’s *t*-test, ** *p* < 0.01). Values were the means ± standard deviation (SD) of three independent biological replicates (*n* = 3).

To assess the efficacy of VIGS in flowers of *H. macrophylla*, the *chalcone synthase* (*CHS*) gene *HmCHS1* was targeted for silencing in stage 2 flowers of ‘Bailmer’ flowers. Phenotypic observations were conducted 15 days post-infection (Figure 3A). Sepals, rather than petals, are the primary ornamental structures of hydrangea flowers. In the TRV-infected flowers, the pink sepals and blue sepals both exhibited darker coloration, while TRV-*HmCHS1*-infected flowers displayed lighter-colored sepals (Figure 3A,B).

RT-qPCR was conducted to confirm the presence of TRV and validate the suppression of *HmCHS1* expression. In the flowers infected with TRV-*HmCHS1*, the expression level of *HmCHS1* was significantly reduced compared to uninfected and TRV-infected flowers (Figure 3C). Furthermore, the total anthocyanin content was notably diminished in the *HmCHS1*-silenced flowers (Figure 3D). These results confirmed the applicability of the VIGS method for functional studies of genes related to flower coloration in *H. macrophylla*.

### 2.4. Functional Validation of the F3′5′H Gene in H. macrophylla

**Figure 4:**
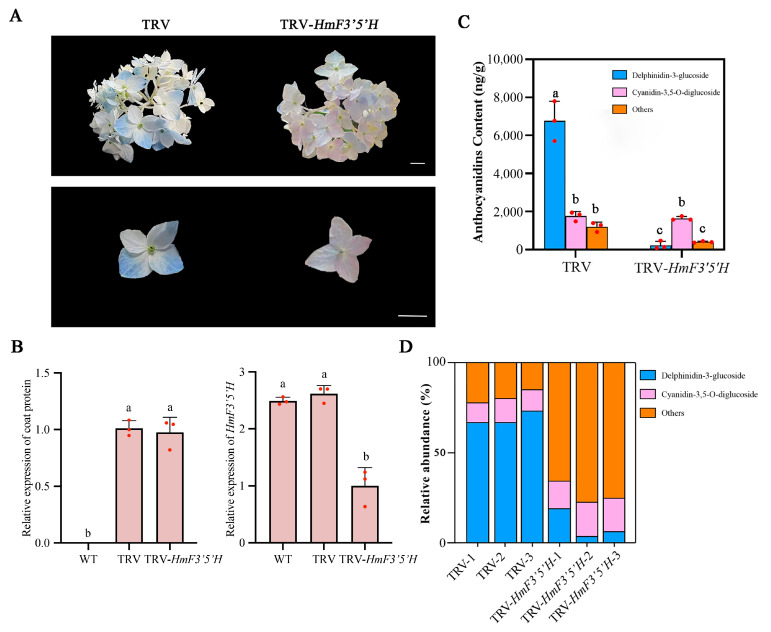
TRV-induced *F3′5′H* silencing in *H. macrophylla*. (**A**) Morphological comparison of TRV-infected and TRV-*HmF3′5′H*-infected flowers at day 15 after infiltration. White scale bar equals 1 cm. (**B**) RT-qPCR analysis to detect relative expression of TRV and *HmF3′5′H* in flowers under various treatments. (**C**) Comparison of the contents of anthocyanidin between *HmF3′5′H*-silenced and TRV-infected flowers. Different lowercase letters indicate significant differences at *p* < 0.05 as determined by Duncan’s test. Values were the means ± standard deviation (SD) of three independent biological replicates (*n* = 3). (**D**) Comparison of the relative abundance of anthocyanidin between *HmF3′5′H*-silenced and TRV-infected flowers. The abundance was the proportion of the certain anthocyanin content in total anthocyanins.

To evaluate the function of the *F3′5′H* gene in anthocyanin synthesis, the TRV-*HmF3′5′H* construct was introduced into stage 2 sepals of ‘Bailmer’ flowers via vacuum infiltration. Phenotypic observations were conducted at 15 days post-infection (Figure 4A). In the TRV-infected flowers, the sepals exhibited blue coloration, whereas in the TRV-*F3′5′H*-infected flowers, the sepals turned pink due to the silencing of *HmF3′5′H* (Figure 4A,B).

RT-qPCR was employed to validate the suppression of *HmF3′5′H* expression. In flowers infected with TRV-*HmF3′5′H*, the expression level of *HmF3′5′H* was significantly decreased compared to the flowers infected with TRV alone (Figure 4B). Furthermore, the content of delphinidin-3-glucoside and total anthocyanidin decreased in *F3′5′H*-silenced flowers (Figure 4C). Delphinidin-3-glucoside was no longer the dominant anthocyanin component in *HmF3′5′H*-silenced flowers.

## 3. Discussion

VIGS technology has been widely employed as a rapid, convenient, and efficient tool for gene functional verification across a variety of plant species [12,13,14,15,16,17,18]. To elucidate significant agricultural traits in *H. macrophylla*, an increasing number of gene sequences associated with various biological processes have been identified, including high-aluminum stress response [19,20], flower induction [21,22], embryo development [23], anthocyanin biosynthesis [20], and blue flower formation [24,25]. Given the challenges associated with stable genetic transformation in hydrangea, developing new techniques to characterize the functions of identified genes has become a pressing issue. In this study, we evaluated the applicability of the VIGS-based silencing system via vacuum infiltration in tissue-cultured seedlings and cut flowers of *H. macrophylla*. Furthermore, the system was successfully employed to validate the functions of the *HmCHS1* and *HmF3′5′H* genes. All data indicate that the TRV-based VIGS protocol was effective for characterizing gene function in *H. macrophylla*.

In order to improve the infection efficiency in tissue-cultured seedlings, vacuum infiltration and syringe injection methods were compared. We found that the traditional syringe injection method was time-consuming and insufficiently effective compared to the vacuum infiltration method. The physiological structure of hydrangea leaves likely affects the entry of the *agrobacterial* mixture. The injection of TRV constructs effectively only a limited area of leaves. Additionally, the syringe injection method was prone to causing mechanical damage to the leaves. Previous studies have demonstrated that the vacuum infiltration method was more efficient compared to other infiltration techniques [26,27]. Consequently, vacuum infiltration of the entire tissue-cultured seedlings was a suitable approach in *H. macrophylla*.

Targeted TRV-induced silencing of the *HmPDS* gene using the vacuum infiltration method in tissue-cultured seedlings resulted in a photobleached phenotype of leaves (Figure 2A), with an infection rate of 60% that was relatively higher than the 34% reported for TRV-*RhPDS* in *Rosa* sp. [8]. Notably, nearly all photobleached leaves exhibited patchy phenotypes, such as white spots or areas of incomplete whitening. We were unable to observe a silencing phenotype in mature leaves, possibly because chlorophyll synthesis in these leaves had ceased. Additionally, the phenotype of *PDS* silencing was particularly pronounced around the leaf veins (Figure 2A). This observation was consistent with previous reports indicating that viral colonization or systemic silencing predominantly occurs in the vascular system [28].

The length of the insert fragment in the viral vector was closely associated with the efficiency of gene silencing. In TRV-infected tobacco, varying lengths of *PDS* insert fragments result in different patterns and extents of photobleaching, and the effective gene lengths of the insert should be in the range of ~200 bp to ~1300 bp [27,29]. In this study, we inserted 300 bp to 400 bp gene fragments into the TRV2 vector, followed by successful silencing of the three genes. Additionally, some factors, including growth temperature and light, also significantly influenced the efficiency of VIGS [9]. In the study, the TRV-*HmPDS* infected plants were shaded for three days and maintained at a slightly lower temperature to facilitate plantlet recovery and the *Tobacco rattle virus* survival.

Flavonoids are crucial metabolites in flowering plants. They are modified to produce various anthocyanins, which confer different colors to flowers. The flavonoid biosynthetic pathway involves the conversion of three molecules of malonyl CoA to chalcone and one molecule of ρ-coumaroyl CoA, catalyzed by chalcone synthase (CHS). Chalcone serves as a precursor for anthocyanin synthesis, undergoing isomerization, hydroxylation, glycosylation, and acetylation to produce various types of anthocyanins [30]. To establish a VIGS system in flowers, sepals were used as the site of infection, and the *HmCHS1* gene related to flower coloration that was identified in our previous study [20] was employed. This experiment confirmed that the *HmCHS1* gene was successfully silenced in hydrangea flowers via the VIGS system.

In the anthocyanin synthesis pathway, F3′5′H and F3′H play essential roles in the production of cyanidin and delphinidin, respectively [31]. Previous studies have shown that delphinidin-3-glucoside in *H. macrophylla* forms blue complexes with Al^3+^ and copigments, imparting a blue color to the sepals [32,33,34,35,36]. Therefore, *F3′5′H* is a key structural gene regulating the formation of blue flowers. In this research, the blue sepals at stage 2 were used for verifying the function of the *F3′5′H* gene. Silencing of the *F3′5′H* gene previously identified [20] led to a decrease in delphinidin-3-glucoside levels; moreover, delphinidin-3-glucoside was no longer the predominant anthocyanin component (Figure 4). Our previous research indicated that the ratio of delphinidin-3-glucoside to total anthocyanins in the sepals was a crucial determinant of the sepal color bluing potential in *H. macrophylla* [36]. Thus, the sepal color in the *HmF3′5′H*-silenced group shifted from blue to pink. Therefore, we conclude that *HmF3′5′H* was a critical gene involved in the synthesis of delphinidin-3-glucoside.

## 4. Materials and Methods

### 4.1. Plant Materials

The cultivar ‘Bailmer’ of *H. macrophylla* was used in this study. Tissue-cultured seedlings were maintained at 25 °C under a light intensity of 2000 lx and a 16 h light/8 h dark cycle. The rooted, tissue-cultured seedlings, which grew to a height of 5–8 cm, were removed from the bottle and precultured in water for one week at 60–80% humidity without altering the culture conditions prior to infection. Cut flowers with the majority of sepals at stage 2 were selected for infection [20].

### 4.2. RNA Extraction

Total RNA was extracted from the upper leaves and sepals of ‘Bailmer’ following the manufacturer’s protocol of the E.Z.N.A^®^ Plant RNA Kit (OMEGA Bio-tek Inc., Doraville, GA, USA). RNA integrity was confirmed via 1.0% agarose gel electrophoresis. Vazyme HiScript II 1st Strand cDNA Synthesis Kit (Vazyme Biotech Co., Ltd., Nanjing, China) was used to synthesize first-strand cDNA following the manufacturer’s protocol.

### 4.3. Isolation and Cloning of HmPDS, HmF3′5′H, and HmCHS1

Phytoene desaturase (PDS) protein sequences from other species were used to screen for homologous sequences in hydrangea using BlasTp based on full-length transcriptome data [20]. The primers used for cloning the open reading frame (ORF) of the *HmPDS*, *HmCHS1* (ON375346.1), and *HmF3′5′H* (ON375346.1) genes are listed in Table 1. PCR was conducted in a 50 μL reaction volume containing 3 μL of cDNA, 25 μL of KOD One^TM^ PCR Master Mix (Toyobo, Osaka, Japan), 18 μL of ddH_2_O, and 2 μL of each forward and reverse primer. The conditions for PCR amplification included an initial denaturation at 98 °C for 2 min, followed by 33 cycles of denaturation at 98 °C for 10 s, annealing at 60 °C for 5 s, and extension at 72 °C for 30 s, concluding with a final extension at 72 °C for 2 min. The purified PCR products were ligated into the cloning vector using the pCloneEZ-Blunt/TA TOPO Cloning Kit (Taihegene, Beijing, China). All cloning plasmids were sequenced by Sangon Biotech Co., Ltd. (Shanghai, China).

### 4.4. Multiple Sequence Alignment

Six PDS homologous protein sequences, which have been identified from grape, tobacco, petunia, tea, and Arabidopsis, were obtained from the NCBI database [14,37,38,39,40,41]. Multiple sequence alignment of *HmPDS* with homologous proteins was conducted with Clustal X 1.81 and BioEdit 7.0 software.

### 4.5. Construction of TRV Plasmids and Agrobacterium Transformation

**Figure 5:**
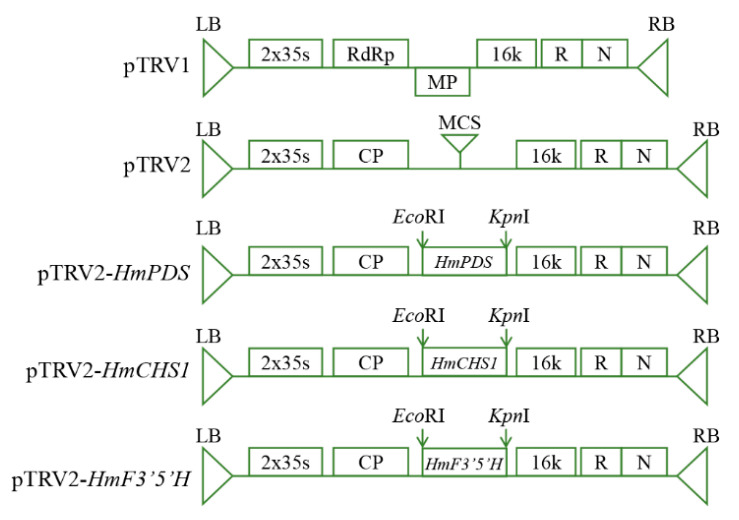
Schematics of pTRV1, pTRV2, pTRV2-*HmPDS*, pTRV2-*HmCHS1*, and pTRV2-*HmF3′5′H*. LB, left border; RB, right border; RdRp, RNA-dependent RNA polymerase; MP, movement protein; 16k, 16 kD protein; R, self-cleaving ribozyme; N, NOS terminator; CP, coat protein; MCS, multiple cloning site.

To generate pTRV2-*HmPDS*, pTRV2-*HmCHS1*, and pTRV2-*HmF3′5′H*, the restriction enzymes *Eco*RI and *Kpn*I (Thermo Fisher Scientific Inc., Waltham, MA, USA) were used to digest the pTRV2 vector. The 382 bp, 332 bp, and 340 bp consensus fragments of *HmPDS, HmCHS1*, and *HmF3′5′H* were PCR-amplified, respectively, using KOD One^TM^ PCR Master Mix (TOYOBO, Shanghai, China) with the primers listed in Table 2. The recombination of the inserted fragments was performed using the ClonExpress^®^ II One Step Cloning Kit (Vazyme Biotech Co., Ltd., Nanjing, China), and the resulting constructs were then transformed into *E. coli* TOP10 (Zhuangmeng, Beijing, China). After overnight cultivation at 37 °C, the presence of inserts was sequenced by Sangon Biotech Co., Ltd. (Shanghai, China). The construction of pTRV2-*HmPDS*, pTRV2-*HmCHS1*, and pTRV2-*HmF3′5′H* vectors is shown in Figure 5. Finally, the successfully constructed vectors were transformed into the *Agrobacterium tumefaciens* strain GV3101 (Zhuangmeng, Beijing, China) using the heat shock method. After overnight cultivation at 28 °C, PCR was performed to identify positive clones.

### 4.6. Virus-Induced Gene Silencing

Positive monoclonal *Agrobacterium* strains containing pTRV1, pTRV2, pTRV2-*HmPDS*, pTRV2-*HmCHS1*, and pTRV2-*HmF3′5′H* plasmids were separately selected and cultured in liquid LB medium with antibiotics (50 mg·L^−1^ kanamycin, 25 mg·L^−1^ rifampicin). The solutions were shaken at 28 °C, 180 rpm, for 16 h, until the optical density (OD_600_) was between 0.8 and 1.2. The *Agrobacterium* cells were harvested by centrifugation (5000× *g*, 6 min) and resuspended to an OD_600_ of approximately 1.5 using the infestation solution (pH 5.6) containing 10 mM MgCl_2_, 10 mM MES, and 200 μM acetosyringone. *Agrobacterium* cultures containing pTRV1 and pTRV2-target gene constructs were mixed in a 1:1 (*v*/*v*) ratio. The culture mixtures were incubated in the dark at 25 °C for 3 to 5 h before treatment.

For VIGS in cut hydrangea flowers, the flowering branches were cut to approximately 5 cm in length in clean water and placed in distilled water for at least 2 h to acclimate to the indoor environment. The plantlets from tissue culture were precultured in deionized water for 7 days. The cut flowers and plantlets were immersed in culture mixtures and then exposed twice to 1 min durations of vacuum (−25 kPa). After the release of the vacuum, the plant materials were cleaned with deionized water and cultured in the dark at 8 to 12 °C for 3 days. After incubation, the cut flowers and plantlets were cultured with a 16 h light/8 h dark cycle at 25 °C for 15 days.

### 4.7. Quantitative Real-Time Fluorescent PCR (RT-qPCR) Analysis

RT-qPCR was used to investigate the expression levels of the target genes in silenced samples. Primers for qRT-PCR were designed using Primer Premier v5.0 software (Premier Biosoft Int., Palo Alto, CA, USA) (Table 3). The specificity and efficiency of the primers were confirmed using 1.5% agarose gel electrophoresis. All reactions were conducted in 96-well plates using the CFX96 Real-Time System (Bio-Rad, Hercules, CA, USA). RT-qPCR analyses were conducted using Taq Pro Universal SYBR qPCR Master Mix (Vazyme, Nanjing, China) according to the manufacturer’s instructions. Each PCR reaction (20 μL) contained 5 μL of cDNA (100–200 ng/μL), 0.4 μL of each primer (10 pmol/μL), 10 μL of 2 × Taq Pro Universal SYBR qPCR Master Mix, and 4.2 μL of ddH_2_O. Amplification reactions referred to previous studies performed [42]. The *coat protein* gene was detected to verify whether TRV had successfully infected the flowers or seedlings [43]. *HmEF1-β* was used as the internal reference gene [44]. The relative expression levels of the target genes were calculated using the 2^−ΔΔCT^ method [45] relative to the internal control.

### 4.8. The Extraction and Measurement of Chlorophyll

Chlorophyll content was measured using a previously established method with some modifications [46]. The upper leaves of the uninfected and TRV-infected plantlets were collected, and each 0.2 g sample was placed in 80% acetone for 24 h to extract chlorophyll. Absorbance values of the extracts at 663 nm and 645 nm were measured using a spectrophotometer (Model 752, Shanghai, China). Three biological replicates were performed for each sample.

### 4.9. The Extraction and Measurement of Total Anthocyanins

Anthocyanins were extracted and quantified using the Plant Anthocyanin Content Assay Kit (BOXBIO, Beijing, China) according to the manufacturer’s protocol. Briefly, 0.1 g of infected sepal samples from the upper flower of the inflorescence were selected, and 1 mL of extraction solution was added. The samples were then ground thoroughly with a mortar and pestle until a homogeneous slurry was obtained. After extraction at 60 °C for 30 min, the mixture was centrifuged at 12,000 rpm for 10 min at room temperature, and the supernatant was collected for measurement. Absorbance was measured at 530 nm and 700 nm using a Multiskan FC enzyme reader (Thermo Fisher Scientific, Waltham, MA, USA). The anthocyanin content was calculated according to the formula provided for 96-well plate determination in the manufacturer’s protocol.

### 4.10. UPLC-PAD Analysis of Anthocyanins in Sepals

Analysis and identification of anthocyanins were performed according to the methods described by Yuan et al. [36]. Sepal samples from upper flowers of inflorescence were ground into a powder in liquid nitrogen. Subsequently, 0.5 g of the sepal powder was extracted for anthocyanin identification. Data were analyzed using Masslynx software version 4.1 (Waters, Milford, MA, USA).

### 4.11. Statistical Analysis

Statistical analyses were performed using SPSS version 17.0 (SPSS Inc., Chicago, IL, USA). All experimental data were tested by a one-way ANOVA or Student’s *t*-test. Figures were generated by GraphPad Prism 8.0.1 software (GraphPad, San Diego, CA, USA).

## 5. Conclusions

In this study, an effective VIGS system based on TRV-*HmPDS* and TRV-*HmCHS1* was established in plantlets and flowers, respectively. The vacuum-infiltration method was successfully employed to silence *PDS* and *CHS* using the TRV vector. Additionally, the function of *HmF3′5′H* in the anthocyanin synthesis pathway in sepals was validated using this technique. Silencing *HmF3′5′H* decreased the contents of delphinidin-3-glucoside, which resulted in a color change in the sepals from blue to pink. The VIGS system developed for *H. macrophylla* will facilitate reverse genetics analyses and exploring genetic resources for molecular breeding.

## Figures and Tables

**Table 1:** Primer sequences for gene cloning.

Gene	Primer Sequence (5′-3′)	Product Size (bp)
*HmPDS*	F:GTGGCAGGGTCAGTATTGCTGG R:CAGACAACGCTTGCCTCAACTAG	1829
*HmCHS1*	F:GGGCACGTGATTCTTAGCTACCAC R:AAGGGACGGAATGCCTCAACTAGACTCG	849
*HmF3′5′H*	F:CACATGTACATACACACAACACTTGCAC R:TCGTGGACGTGGTTGAATGG	1594

**Table 2:** Primer sequences for pTRV2 vector construction.

Gene	Primer Sequence (5′-3′)	Product Size (bp)
*HmPDS*	F: CTGTGAGTAAGGTTACCGGCTATGCCAAACAAGC R: GAGACGCGTGAGCTCGCGGAGGATTACCATCTAA	382
*HmCHS1*	F: CTGTGAGTAAGGTTACCGATGGTGACCGTCGAGG R: TCGAGACGCGTGAGCTCGGTGGCTGCTTCTTTGCCTAG	332
*HmF3′5′H*	F: CTGTGAGTAAGGTTACCGCCGAGACCCGGATGTTTG R: TCGAGACGCGTGAGCTCGCGTGGACGTGGTTGAATG	340

**Table 3:** Primer sequences for RT-qPCR.

Gene	Forward Primers (5′-3′)	Reverse Primers (5′-3′)
*HmEF1-β*	CGCAGCTGTTTTAGGGAAGCC	GCGAGCTGCGAAGACACAGA
*Coat Protein*	TTACGACGAACCAAGGGAGTACTA	CGGTGCAGATGAACTAGCAGCTG
*HmPDS*	GCCAATGCAATGCAATGAGCTG	GGCAGACATCCATCCTTGGTCTTG
*HmCHS1*	AATTTCAGCGCATGTGTGACAATT	CCACCACCATGTCTTGTCTAGC
*HmF3′5′H*	AGGGCAAGCCGGACTTTCTT	CCGGCAGTGAACAAATTCAAGAGTA

## Data Availability

Data will be made available upon request.
